# Reduced Intraoperative Blood Loss and Hypothermia in Burn Surgery using Cardiopulmonary Bypass Pumps

**DOI:** 10.1177/22925503211024744

**Published:** 2021-06-16

**Authors:** Amit Persad, Kevin Mowbrey, Edward Tredget

**Affiliations:** 1 Division of Neurosurgery, Department of Surgery, University of Saskatchewan, Saskatoon, SK, Canada; 2 Division of Plastic Surgery, Department of Surgery, University of Alberta, Edmonton, Alberta, Canada

**Keywords:** TBSA >40, roller pump, insufflation, transfusion, intraoperative normothermia

## Abstract

**Objective:** Patients presenting with total body surface area (TBSA) >40% burns require significant surgical treatment. Two substantial challenges during these surgeries are limiting blood loss and maintaining core temperatures. To overcome these challenges, several techniques have been developed, ranging from the Pitkin syringe method to the pneumatic tourniquet strategy for large-volume hyperthermic insufflation. Here, we compare the pneumatic tourniquet method to a roller pump method for maintenance of intraoperative normothermia and control of bleeding. **Methods:** We conducted a retrospective chart review of 20 patients presenting with TBSA >40% burns, 10 of whom were treated with the rapid infusion roller pump and 10 of whom were treated with the pneumatic tourniquet technique. Patients from each group were controlled for % TBSA, presence of inhalation injury, age, and date of admission. We reviewed transfusion requirement and the intraoperative temperatures, as well as the average intraoperative drop in temperature. **Results:** We observed improvement in the infusion volume, operative time, intraoperative temperature drop, minimum intraoperative temperature, estimated blood loss, and amount of required transfusion. **Conclusions:** Our study suggests that the rapid infusion roller pump technique is capable of achieving superior intraoperative bleeding control and temperature maintenance compared to the pneumatic tourniquet technique, resulting in decreased transfusion requirement.

## Abstract (fr)

**Objectif:** Les patients ayant des brûlures sur plus de 40% de la surface corporelle totale (SCT) nécessitent un traitement chirurgical significatif. Ces interventions chirurgicales sont confrontées à deux défis majeurs: la limitation des pertes sanguines et le maintien de la température corporelle. Pour vaincre ces défis, plusieurs techniques ont été développées, allant de la méthode de la seringue de Pitkin à la stratégie du garrot pneumatique pour insufflation hyperthermique de grand volume. Nous comparons ici la méthode du garrot pneumatique à la méthode de la pompe à galets pour le maintien de la normothermie peropératoire et le contrôle des saignements. **Méthodes:** Nous avons mené une étude rétrospective des dossiers de 20 patients présentant des brûlures sur > 40 % de la SCT; 10 d’entre eux ont été traités avec la pompe à galets pour perfusion rapide et 10 ont été traités avec la technique du garrot pneumatique. Les patients de chaque groupe ont été contrôlés pour le pourcentage de SCT, la présence de lésions d’inhalation, l’âge et la date de leur hospitalisation. Nous avons revu les besoins en transfusion ainsi que les températures peropératoires et la chute moyenne peropératoire de la température. **Résultats:** Nous avons observé une amélioration du volume de perfusion, de la durée d’intervention, de la chute peropératoire de la température, de la température peropératoire minimum, de la perte sanguine estimée et de la quantité requise de transfusion. **Conclusions:** Notre étude suggère que la technique de perfusion rapide avec une pompe à galets permet d’obtenir un meilleur contrôle peropératoire des saignements que la technique avec garrot pneumatique, aboutissant à une diminution des besoins de transfusions.

## Introduction

Current treatment for extensive burns spanning >40% total body surface area (TBSA) is limited by hemodynamic and thermoregulatory status during extensive debridement. During burn resuscitation and operative treatment, volume status and circulatory collapse are major causes of morbidity and mortality. Prior to resuscitation, circulatory status is threatened in extensive burn injuries, with microvascular damage and associated inflammatory changes and distal edema associated with burns >20% TBSA.^1,2^ During burn surgery, factors such as evaporative volume loss due to the loss of the protective epidermal layer of the skin also contribute to fluid loss. In order to minimize blood loss and the amount of transfusion, which is associated with increased rates of infection and mortality,^3,4^ methods such as subeschar and donor site clysis^5-7^ as well as epinephrine instillation^7^ have been developed for more effective control of blood loss, most particularly using a tumescent technique. Furthermore, large volume fluid resuscitation in combination with compromise of the epidermal layer^8^ leads to increased risk of intraoperative hypothermia, which is associated with blood loss, need for transfusion, and mortality rates in burn victims.^9-11^

Current methods have evolved to reflect this need for high rate insufflation of burns. In the past, the manually driven “Pitkin” syringe was used for intraoperative clysis^10^ before the introduction of the more efficient pneumatic tourniquet (PT) method,^5^ used in the majority of cases at our site prior to 2006. The major limitations of this technique include replacement of solution bags, limited flow rate, and lack of thermal control of the infused solution. More recently, we are using the Cobe roller pump (RP) system, which offers an increased degree of control over infusion temperature as well as pump pressure and rate.

The PT system^5^ is performed using a combination of a PT, arterial pressure bags, and spinal needles for insufflation. The principle of the RP technique is similar, but instead of the PT and pressure bags uses a motorized RP to provide the forward pressure for insufflation. Both techniques use warmed saline and spinal needles for introduction of clysis.

The RP system coupled with heat exchanges increases the maximum clysis volume as well as improves intraoperative thermoregulation, leading to decreased intraoperative hypothermia. Furthermore, the enhanced delivery of epinephrine to wound sites improves intraoperative control of bleeding and thus decreases transfusion-associated complications.

Here, we report our experience of using the rapid infusion RP device, which holds advantages over the more traditional PT system in dramatically increased clysis volume, decreased intraoperative temperature change, decreased blood loss, and less transfusion requirement.

## Methods

**Figure 1.**
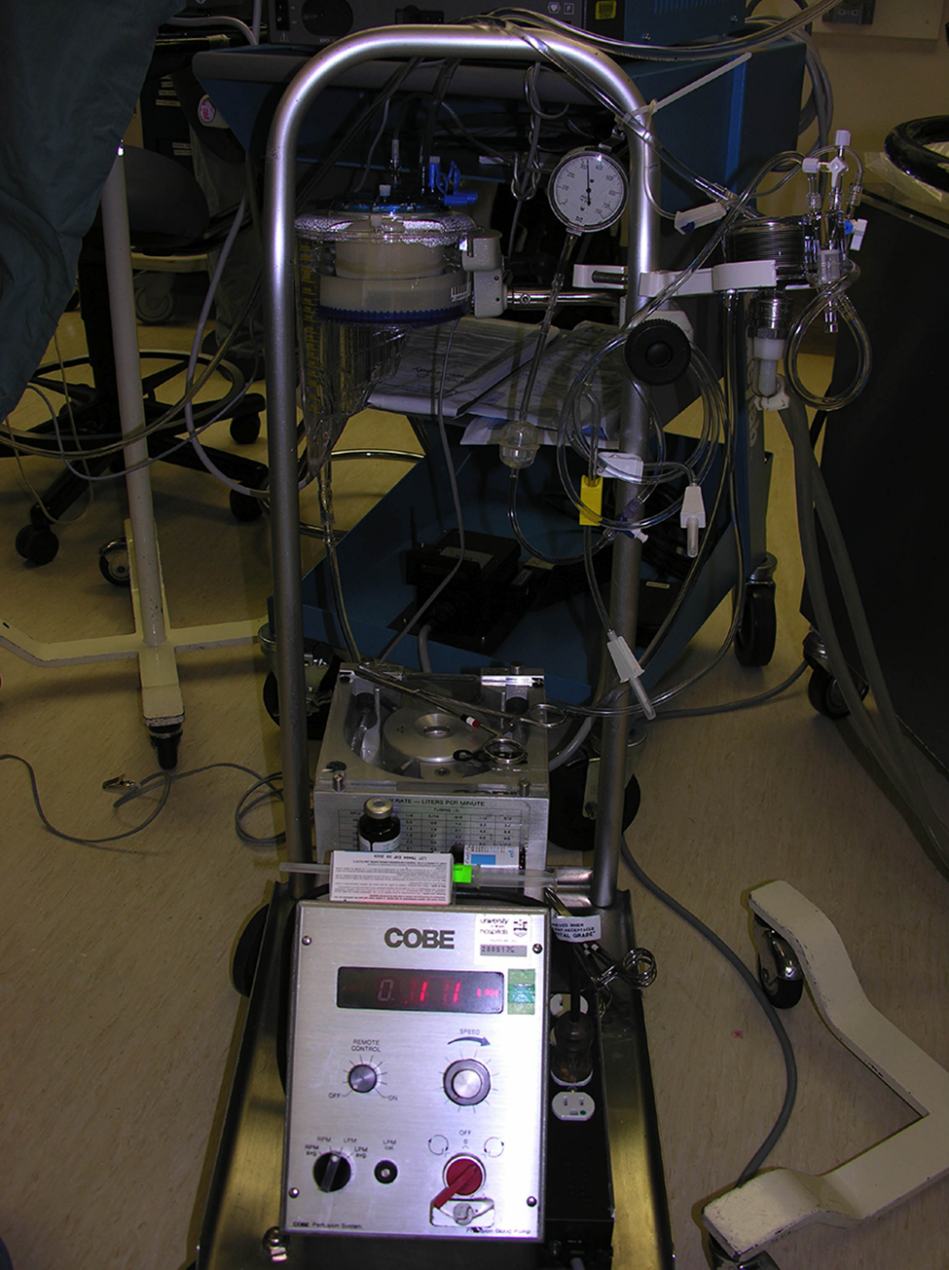
A photograph of the roller pump system, which is composed of a Cobe Roller Pump (Stockert 10-00-00 Roller Pump, item number 273671016507), Sarns Heater Cooler Unit, Medtronic Myotherm XP Cardioplegia Delivery Device with Heat Exchanger, Dideco Midicard D764 reservoir, a 3/16″ × 1/16″ Delivery Line, Medtronic Multiperfusion Set, High Flow Extension Line, 18 Ga Spinal Needles, and 30-in extension set, double male connector pressure manometer.

**Figure 2.**
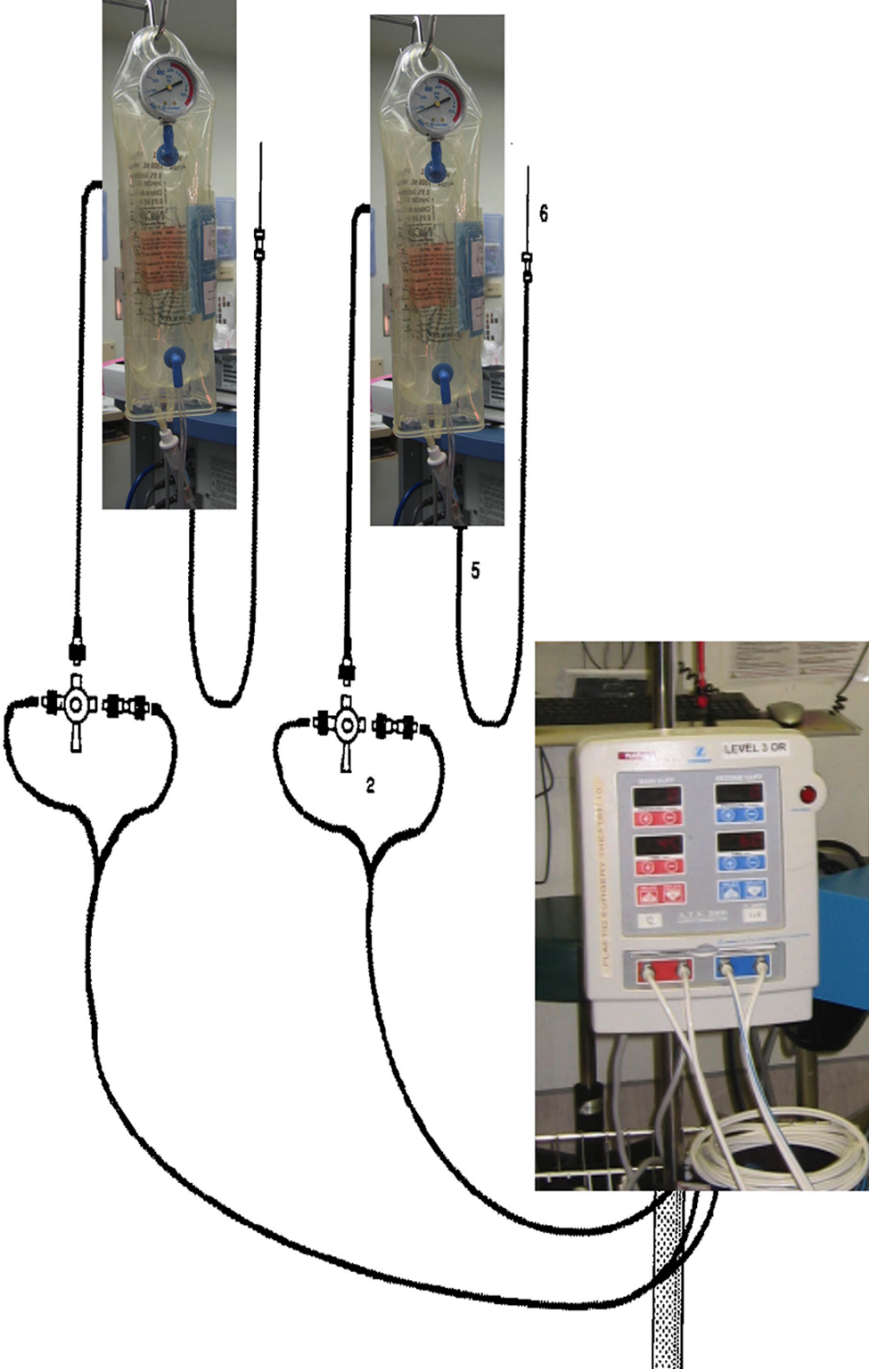
Graphical representation of the pneumatic tourniquet system,^5^ which serves as a control in this study.

We conducted a retrospective chart review comparing cohorts of patients who were treated for burns with TBSA >40% between 2004 and 2010. All patients were operated on by a single surgeon at 1 institution, each time with another burn surgeon as an assistant. Patients having undergone surgery with clysis via the RP system (Figure 1) between 2006 and 2010 (n = 10) were compared with patients having undergone clysis with the PT (Figure 2) system between 2004 and 2006 (n = 10).^5^ Patients operated on prior to 2006 had clysis with the PT system, and after 2006 had clysis using the RP system. The epinephrine used is 1:400,000 concentration.

Inclusion criteria included age >18 years, TBSA>40%, and requirement for debridement without repositioning. Rates of inhalational injury were similar between groups. The presence or absence of inhalation injury is documented by clinical examination, bronchoscopy, chest X-ray, and arterial blood gases.^11,12^

The operative rapid insufflation system used was comprised of the Cobe Roller Pump (Stockert 10-00-00 Roller pump, item number 273671016507), Sarns Heater Cooler Unit, Medtronic Myotherm XP Cardioplegia Delivery Device with Heat Exchanger, Dideco Midicard D764 reservoir, a 3/16″ × 1/16″ Delivery Line, Medtronic Multiperfusion Set, High Flow Extension Line, 18 Ga Spinal Needles, and 30-in extension set, double male connector pressure manometer. The technical specifications of this system are outlined: 7.5 mL epinephrine 1:1000 per 3 L normal saline (2.5 mg/mL epinephrine as per institutional standard); insufflation solution temperature is maintained at 40 °C, utilizes multiperfusor, high flow extension set, and 4 spinal needles; achieves insufflation pressures of 300 to 500 mm Hg; and pump flow rates of ∼200 to 300 mL/min. Clysis using the RP system was carried out in accordance with the tumescent technique. Spinal needles were directly introduced to burn sites for insufflation in the subcutaneous tissues. Following insufflation, escharotomy, debridement, tangential excision, removal of granulation tissues, and skin grafting were carried out in the usual fashion. We used underbody heaters, level 1 warmers for intravenous fluids, and ambient lights for all patients. Pneumatic tourniquet technique was carried out as previously described.^5^

**Figure 3.**
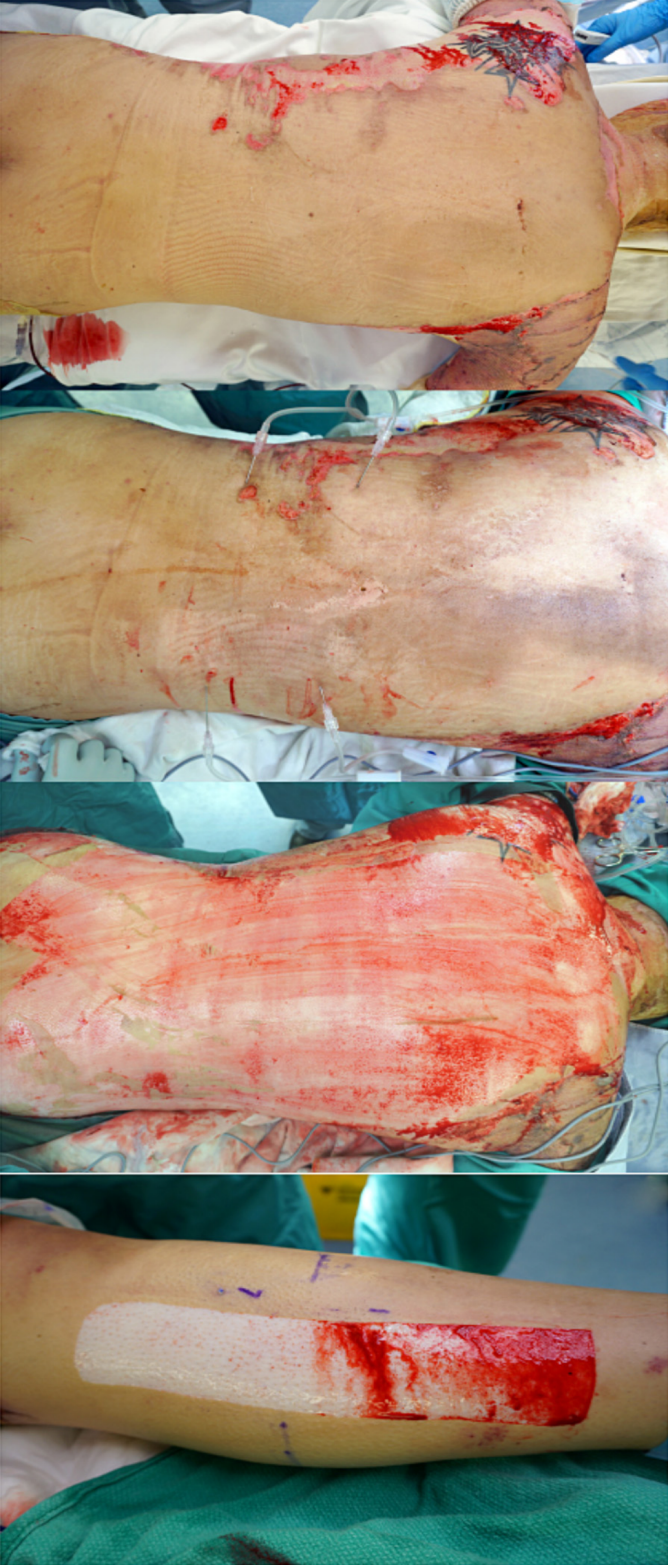
Photograph of insufflated donor sites using roller pump system.

All patients included had TBSA>40%, no need for repositioning during surgery, and all patients had debridement, tangential excision, and skin grafting at minimum. Anatomical locations of debridement included anterior torso and limbs. No patients with facial or neck burns were included. Skin grafts were meshed following harvesting. Limb tourniquets were used for both techniques. Donor sites were insufflated until tissues were palpably firm (Figure 3). We find that this technique makes the technical aspect of harvesting the graft easier by making the skin less mobile.

Blood transfusion was measured intraoperatively and was at the discretion of the anaesthesiologist. Donor sites were included in the estimates of overall blood loss and did impact the amount of blood that was transfused by the anaesthesiologists.

No patient had postoperative bleeding.

Insufflation volume, operative time, operative area debrided, maximum intraoperative temperature drop, minimum intraoperative temperature, estimated blood loss, and volume of intraoperative blood product transfusion were extracted from chart records. The average temperature drop was computed as the difference between preoperative holding temperature and the lowest recorded intraoperative temperature. Clinical estimations of blood loss recorded in the charts were used for analysis. The data were averaged for cohorts and compared via Student *t* test. Data are reported as mean ± standard deviation.

## Results

Characteristics of included patients were similar between the PT control and RP groups, with age (PT: 39.7 ± 5.1, RP: 42.4 ± 4.7, *P* = .74), burn TBSA (PT: 48.1 ± 1.4, RP: 49.7 ± 3.6, *P* = .56), number of operations per patient (PT: 3.2 ± 0.6, RP: 2.9 ± 0.6, *P* = .83), and number of procedures per cohort (PT: 31, RP: 37) being the controlled parameters (Table 1). Shapiro-Wilk test demonstrated normality of the data (PT: *P* = .74, RP: *P* = .63).

**Table 1.** Controlled Parameters of Included Patients, Including Age, % TBSA, Procedures Per Patient, and Total Procedures Per Group Are Summarized.

Variable	Pneumatic tourniquet (PT)	Roller pump (RP)	*P* value
Age	39.7 ± 5.1	42.4 ± 4.7	.74
TBSA (%)	48.1 ± 1.4	49.7 ± 3.6	.56
Procedures per patient	3.2 ± 0.6	2.9 ± 0.6	.83
Total procedures	31	37	

Abbreviation: TBSA, total body surface area.

The operative area debrided was similar between groups (PT: 2785 ± 321 cm^2^, RP: 2868 ± 249 cm^2^, *P* = .71). Shapiro-Wilk test demonstrated normality of the data (PT: *P* = .51, RP: *P* = .94). Average insufflation volume using the RP system far outstripped the PT system (PT: 3059 ± 691 mL, RP: 7570 ± 859 mL, *P* = .04), with an average of 18.9 mg of epinephrine delivered with the RP system. Shapiro-Wilk test demonstrated normality of the data (PT: *P* = .66, RP: *P* = .71). The operative time was slightly less for patients undergoing clysis via the RP system (PT: 239 ± 13 minutes, RP: 204 ± 11 minutes, *P* = .03). Shapiro-Wilk test demonstrated normality of the data (PT: *P* = .77, RP: *P* = .91).

Thermoregulation using the RP technique was also superior, indicated both by lesser average maximum temperature drop (PT: 2.05 ± 0.41 °C, RP: 1.00 ± 0.16 °C, *P* = .04), with Shapiro-Wilk test demonstrating normality of the data (PT: *P* = .94, RP: *P* = .87); and by higher minimum intraoperative temperature (PT: 35.24 ± 0.23 °C, RP: 36.01 ± 0.22 °C, *P* = .04), with Shapiro-Wilk test demonstrating normality of the data (PT: *P* = .78, RP: *P* = .81).

**Figure 4.**
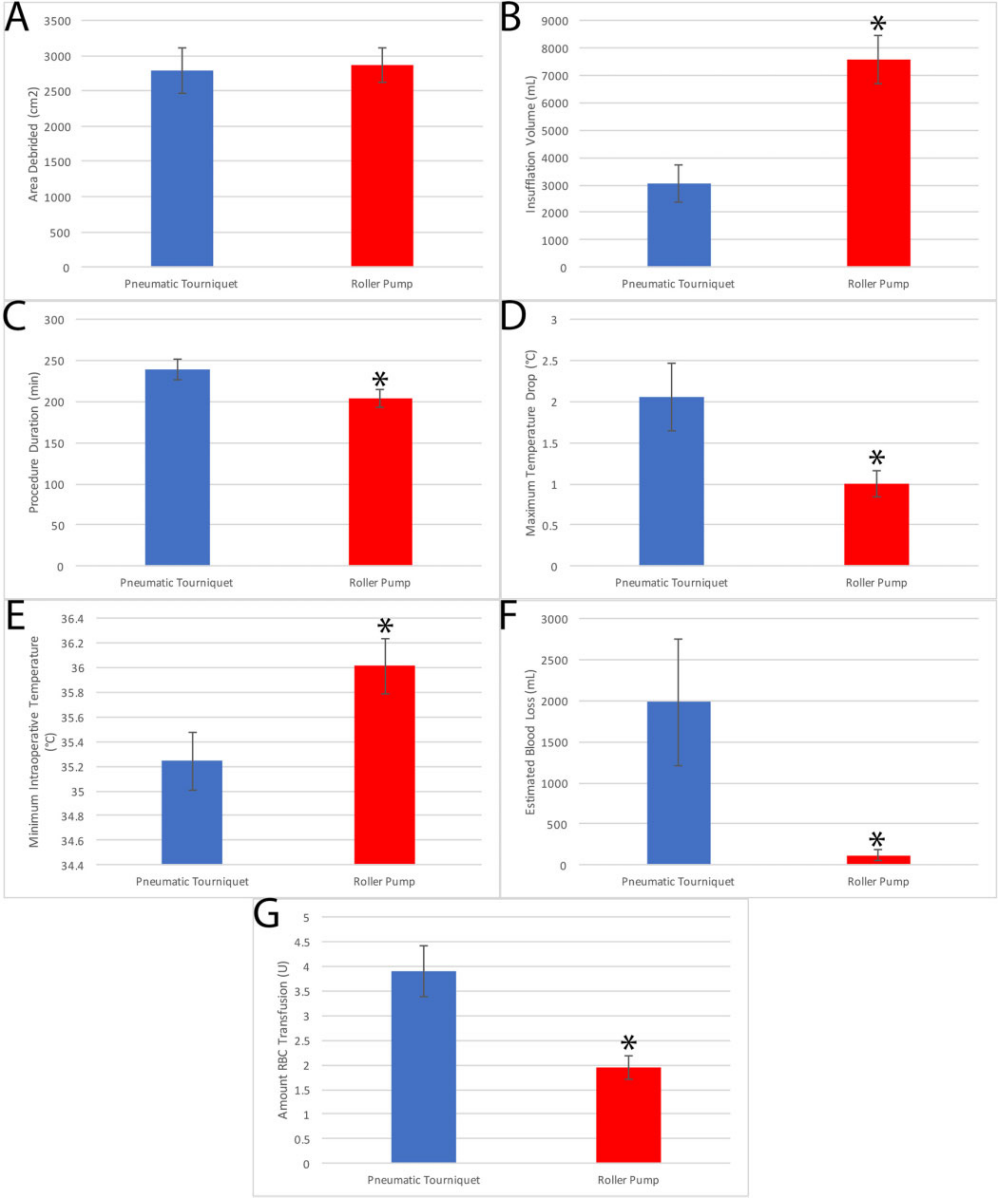
Results of Table 2 in graphical format. A, Area debrided, (B) insufflation volume, (C) procedure duration, (D) greatest intraoperative temperature decrease, (E) lowest intraoperative temperature, (F) amount of blood loss, and (G) units of blood transfusion. Blue: pneumatic tourniquet and Red: roller pump. * denotes *P* < .05.

Finally, the RP technique achieved superior control of bleeding to the more traditional PT system, averaging less blood loss (PT: 1981 ± 768 mL, RP: 121 ± 74 mL, *P* = .02), with Shapiro-Wilk test demonstrating normality of the data (PT: *P* = .72, RP: *P* = .80). We also had less transfusion of blood product intraoperatively (PT: 3.90 ± 0.51 U, RP: 1.94 ± 0.23 U, *P* = .03) per surgery, with Shapiro-Wilk test demonstrating normality of the data (PT: *P* = .91, RP: *P* = .88). Results are summarized in Table 2 and displayed graphically in Figure 4.

**Table 2.** Results of Study.^a^

Variable	Pneumatic tourniquet (PT)	Roller pump (RP)	*P* value
Area debrided, cm^2^	2785 ± 321	2868 ± 249	.71
Insufflation volume,^b^ mL	3059 ± 691	7570 ± 859	.04
Procedure duration,^b^ min	239 ± 13	204 ± 11	.03
Maximum temperature drop,^b^ °C	2.05 ± 0.41	1.00 ± 0.16	.04
Minimum intraoperative temperature,^b^ °C	35.24 ± 0.23	36.01 ± 0.22	.04
Estimated blood loss,^b^ mL	1981 ± 768	121 ± 74	.02
Amount RBC transfusion,^b^ U	3.90 ± 0.51	1.94 ± 0.23	.03

Abbreviation: RBC, red blood cell.

^a^ Parameters observed include area debrided, insufflation volume, procedure duration, maximum temperature drop, minimum intraoperative temperature, estimated blood loss, and blood products transfused.

^b^ Statistical significance (*P* < .05).

No patients had hemodynamic instability secondary to epinephrine administration, arrhythmias, or cardiac events intraoperatively or postoperatively.

## Discussion

Blood loss is the major factor limiting effective surgical treatment of burns patients^6^ due in part to the nature of tangential excision. Current evidence suggests that methods including insufflation of the injured site with epinephrine improve control of bleeding via vasoconstriction.^6,13^ Other factors such as intraoperative hypothermia^14^ have adverse effects on total blood loss and transfusion requirement. High flow clysis is essential to burn management both for the delivery of local vasoconstrictors and insufflation of the wound as well as for contouring of incision sites at the burn surface and at donor graft sites. Furthermore, unlike other available methods, the RP technique is able to continuously supply clysis to surgical sites, unlike more traditional bolus instillation methods, without the need for continuous replacement of fluid bags as in the PT method. However, replacement of bags might be useful to ensure adequate warming of fluid.

Of note, patients treated using the RP technique had superior thermal control and reduced decrease in body temperature intraoperatively. Similar benefits to thermoregulation have been previously described in the setting of tumescent clysis using epinephrine solutions. Many approaches to intra- and perioperative thermoregulation have been described,^15,16^ including passive as well as forced air warming and fluid garment warming.^16,17^ Circulating water garments have been shown to be more effective than the other commonly used methods. Intraoperative thermoregulation using an intravascular approach has also been described as an adjuvant approach,^16-18^ although its use is relevant to an intensive care unit setting and not for burn surgery.

Potential for adverse outcomes does exist using the RP method. Because of the high flow rate of clysis used, fluid overload or adrenergic complications from large dose infusion of epinephrine may occur. We do not report any incidence of these adverse outcomes within our cohort. Intraoperative thermoregulation is an important challenge for larger surface area burn surgery, as the destruction of the dermal barrier reduces the ability of the body to thermoregulate. Hypothermia is a limiting factor in the operating room, and better temperature control may therefore allow for longer operations.

One issue with the usage of the RP technique is the high volume of insufflated epinephrine. The reported maximum dosage of subcutaneous epinephrine in adults is 7 to 8 mg, while 4 mg is the minimum reported fatal dose.^19^ The mean dose of epinephrine delivered by the RP system was 18.9 mg. High doses of subcutaneous epinephrine and lidocaine have been reported within the liposuction literature, which is possible as a large amount of the insufflated epinephrine is removed during the suction of the subcutaneous tissues.^20^ It is possible that a large volume of clysis is removed during burn surgery as well due to extensive tangential excision procedures overlying the sites of epinephrine insufflation, although the clysis location is subdermal and so the insufflated volume may not be released. In addition, because of vasoconstriction secondary to epinephrine, the systemic absorption of epinephrine is itself limited. The absorption of epinephrine depends on the site of injection as well, with the face and neck often leading to more rapid absorption. No arrhythmias, tachycardia, or hypertension, which are the common major adverse events associated with epinephrine administration, were encountered within either patient group.^21,22^ We used 1:400 000 epinephrine in saline which is considered the maximum dilution of epinephrine solution that retains its vasoconstrictive benefits to control blood loss.^23^

The use of epinephrine has been shown to lead to improvement in outcomes in burn surgery for both tangential excision and for graft harvest, with reduced intraoperative blood loss and transfusion requirement.^24-28^ In addition, the use of epinephrine in graft harvest has been shown not to lead to any poor outcomes compared with plain lidocaine.^29^ We show results concordant with these effects, with massive reduction of intraoperative bleeding.

Other methods for tumescent infiltration of burn sites have been described using syringes and pressurized fluid bags. We describe the use of an RP, which leads to increase instillation pressure and the ability to continuously infiltrate tissues. A reduction in total procedure duration in the RP group reflects these features, as clysis to burn sites using the RP technique is quick due to the pressurized fluid delivery. In addition, the technique allows the operator to simultaneously insufflate new areas and perform debridement on other, already insufflated areas.

The RP is not novel but the use of the heart-lung bypass RP attached to the countercurrent heating device is not described in the literature. The difference is the ability to heat the fluid being sufflated which has had a major impact on the development of hypothermia during the burn cases. The larger RP more rapidly infuse fluid leading to more complete insufflation and less blood loss, as well as shorter duration of surgery. A number of burn centres continue to use and describe Pitkin syringes to achieve insufflation which is very slow, incomplete, and hypothermic in terms of the fluid insufflated. Other centres that use the RPs alone and do not derive the benefit of the warmed fluids for patients with burn undergoing large and long debridements. We suggest that the use of the RP warms the fluid and most importantly reduces temperature loss in the operating room and hypothermia. The more rapid pump gives more complete insufflation and less blood loss.

The main limitation of this study is sample size, as only 10 patients were analyzed in each group. Regardless, statistically significant changes in the mentioned variables, particularly thermoregulation, control of blood loss, and transfusion volume, were noted. Further investigations comparing large groups of patients with burn being treated using both methods are needed in order to fully characterize the benefits of using the RP system. We used Shapiro-Wilk test of normality in order to facilitate statistical comparison, but ideally a study with larger sample size would be performed in the future.

The study was retrospective and estimates for blood loss from each operative report were made years before the study was undertaken. Although the RP was used in a later time period than the PT, the same surgeon was involved where not much difference in experience changed to alter the rate of the surgery. Similarly, our approach to transfusion was the same in the 2 time periods although we now restrict the amount of blood given as described in the following manuscript. The liberal approach to transfusion in the manuscript was employed during the periods in which the study was conducted.^30^

## Conclusion

By using the RP method when treating patient with extensive burns >40% TBSA, more effective control of bleeding and core temperature control may be achieved through high output clysis with thermoregulated fluid. This represents an important improvement in surgical management of patients with burn, which may improve patient outcomes, associated with excessive bleeding and hypothermia as well as adverse outcomes of overtransfusion. Assessment of this technique for longitudinal outcomes may yield insight into the efficacy of this technique in averting serious adverse outcomes of burn surgery, such as circulatory collapse, myocardial events, and sepsis, as well as assessing the risk of adverse outcomes associated with the RP method.
